# Current Knowledge of Respiratory Function in Early Onset Scoliosis and the Effect of Its Contemporary Surgical Treatment

**DOI:** 10.3390/jcm15020754

**Published:** 2026-01-16

**Authors:** Sai Gautham Balasubramanian, David Fender, Paul Rushton

**Affiliations:** Great North Children’s Hospital and Royal Victoria Infirmary, Newcastle Upon Tyne NE1 4LP, UK; sai.balasubramanian1@nhs.net (S.G.B.); d.fender@nhs.net (D.F.)

**Keywords:** Early Onset Scoliosis, pulmonary function, Growing Rods, MCGR, VEPTR, growth guidance, PFT

## Abstract

Early Onset Scoliosis (EOS), defined as presenting before 10 years of age, often has a significant adverse impact on pulmonary function, due to a complex interrelationship between the spine, chest, pulmonary structures and their development. Left untreated, EOS leads to premature death, with early fusion surgery to arrest curve progression making little impact on this. To date, the natural history has not been clearly established as compounded by the heterogeneity of pathologies, causing EOS and challenges in objective measurements of pulmonary function in this young age group. A desire to address this poor natural history has motivated interest in pursuing ‘growth friendly’ surgical strategies. The implants used have evolved with time, often to address compromises and poor results, with multiple options now available based on treatment principles (distraction, compression, or guided growth systems). The aims of such strategies are to control the structural spinal deformity, whilst allowing spinal and thoracic growth, with the seemingly reasonable expectation that this will result in improved pulmonary function and avoidance of premature death. Most studies have focused on radiological outcome measures such as Cobb angle and thoracic height to gauge the success of surgery, with these measures acting as surrogate markers of improved pulmonary outcome. This assumption, however, is not supported by more recent clinical data which has attempted to assess directly the pulmonary outcomes associated with growth-friendly surgical strategies. This literature review therefore sets out to characterise the effect of EOS on pulmonary function and to critically analyse the impact surgical treatment options will have while addressing this.

## 1. Introduction

Early Onset Scoliosis (EOS) is defined by the Scoliosis Research Society as scoliosis presenting before age 10 years. It is a description rather than a diagnosis, encompassing scoliosis of all aetiologies. The function and outcome of this heterogeneous group are heavily influenced by the underlying diagnosis. Children with idiopathic scoliosis, being otherwise well, allow us to assess the influence of spine deformity on respiratory function with greater clarity. Pehrsson et al. [1], in examining the effects of progressive idiopathic scoliosis, highlight the importance of age of onset. Children with onset within the first three years of life (infantile) had significantly increased risk of premature death compared to the expected rate, largely due to respiratory failure. This effect was present, but less striking, in those with scoliosis onset between 4 and 9 years (juvenile), but was not seen when scoliosis occurred at age 10 or more. These stark findings are supported by others, identifying significantly worse pulmonary function tests in those with an onset of idiopathic scoliosis at <5–6 years old [2,3]. Branthwaite [2] reported 10/15 children with infantile onset scoliosis developed respiratory failure before the age of 60. Similarly, of 28 children with infantile idiopathic scoliosis followed up for 12 years in Oxford, 4 had died, including 3 from cardio-respiratory failure [4]. The association of Early Onset Scoliosis with cor pulmonale is also well recognised [5].

Such data have shifted the focus for treatment of Early Onset Scoliosis away from solely correcting spinal shape to maximising lung function at the end of growth. The aim of looking after these children is to ensure that they have sufficient respiratory function at skeletal maturity to avoid respiratory failure and premature death due to the natural deterioration in function throughout adult life [6,7,8].

Whilst there are broader functional and quality of life benefits to be gained from surgery, addressing respiratory dysfunction remains the primary goal given the acknowledged poor natural history. This complex topic crosses medical specialities, with the literature developing steadily over the last 40 or so years. There are few detailed reviews particularly covering the aetiology, pathophysiology, and natural history of chest function in EOS in significant depth. We have, therefore, reviewed this literature with the following aims:

1. Characterise the effect of EOS on respiratory function.
2. Establish the effect of contemporary surgical treatment of EOS on respiratory function.

## 2. Methodology

A comprehensive search was performed in the PubMed, Scopus, and Web of Science databases following established guidelines for systematic reviews using the following keywords: “Early Onset Scoliosis”, “Growth friendly techniques”, “Pulmonary function tests”, “Respiratory function”, and “Pulmonary outcome.”. Studies were identified to address the aforementioned aims of the paper. Given the literature identified, a narrative approach was taken.

## 3. The Effect of EOS on Pulmonary Function

### 3.1. Evaluation of Lung Function

Whilst appreciating the compelling mortality data, measurement of the effect of EOS on respiratory function is challenging with most children too young to comply with traditional pulmonary function testing. This has led to a paucity of data both for operated children but also crucially untreated children. This lack of data regarding the natural history of EOS on respiratory function leaves surgeons with little to guide them when embarking on treatment and its respiratory aims.

When objective measurements are absent, surgeons should be cautious using traditional 2D measures (Cobb angle) of spinal deformity as surrogates for respiratory function, as the correlation between PFTs and Cobb angle are moderate at best [3,9,10,11,12]. Similarly, whilst asymmetrical lung perfusion and ventilation affect around half of those with EOS, the side with reduced function does not appear to have a predilection for the convex/concave lung or the asymmetry related to Cobb angle [13]. Furthermore, clinical evaluation of respiratory status is insensitive with children unlikely to be short of breath until their Forced Vital Capacity (FVC) falls to <50% predicted. Functional tests, such as the 6 min walk test, designed to assess aerobic capacity and endurance, have shown reduced performance in those with congenital EOS compared to normal children, but do not correlate with percentage predicted FVC [14].

#### 3.1.1. Natural History

In a rare series of largely untreated children with EOS examined as teenagers, Owange-Iraka et al. [9] identified significantly reduced Vital Capacity in those with Early Onset Scoliosis compared to predicted values. Of 30 children with infantile idiopathic scoliosis, the mean reduction in Vital Capacity (VC) was 47% at mean age 15. Congenital scoliosis had a greater impact, with a mean reduction in VC of 53% at mean age 13 years, perhaps related to associated factors such as rib abnormalities [9]. In a cross-sectional study, Redding and Mayer [10] examined sedated infants (mean age 15 months) and discerned that 5 of their 10 cases with infantile onset scoliosis had an FVC at least one standard deviation below their predicted value. Other studies have consistently confirmed the largely restrictive deficit seen in EOS with associated reduction in total lung capacity (TLC), VC, and forced expiratory volume in 1s (FEV1) [11,15], whereas the effect on residual volume is conflicting [11,12]. Overnight sleep studies have also been used in a young group with thoracic insufficiency syndrome, identifying that the vast majority have sleep disordered breathing including apnoeic episodes and desaturations [16].

#### 3.1.2. Impact

The impact of reduced respiratory function is better understood. Children with poorer lung function work harder with higher respiratory rates, expending more calories whilst commonly finding eating tiring, reducing their calorific intake and resulting in failure to thrive [17]. Severe Early Onset Scoliosis, especially if associated with associated thoracic congenital abnormalities, may result in thoracic insufficiency syndrome (TIS). This is defined as the inability of the thorax to support normal respiration or lung growth [18]. Campbell et al. [18] considered normal respiration as characterised by a normal respiratory rate, effortless breathing, age-appropriate exercise tolerance, and normal arterial oxygen levels. Children treated for thoracic insufficiency syndrome not only had worse physical function domains scores compared to unaffected children but they also scored more poorly than those with asthma, epilepsy, heart disease, and childhood cancers [19].

### 3.2. The Effect of EOS on Pulmonary Anatomy

In the simplest terms the respiratory system consists of lung tissue, supplying airways and chest cavity. Early Onset Scoliosis may affect all facets of this.

#### 3.2.1. Lung Tissue and Supplying Airways

Gas exchange occurs at the alveolar level; thus, the number and size of alveoli and their blood supply is fundamental to lung function. The work of Dunnill informed the established view that alveolar numbers increase exponentially from birth to around age 8 years, with lung expansion over the remainder of growth occurring through enlargement of this established lung tissue [20]. Others describe alveolarisation as largely complete by 2 years, but ongoing septal restructuring with microvascular remodelling occurrs until age 6 [21]. More recent MRI studies suggest alveolar numbers as well as size may increase, at least to some extent, into adolescence [22].

Whilst acknowledging these uncertainties regarding lung development, it is agreed clinically that lung function is most adversely affected by the development of significant scoliosis during the first 5 or so years of life. Rabbit models demonstrate that onset of scoliosis at a rabbit age analogous to a 3-year-old child leads to pulmonary hypoplasia, reduced lung volume, and compliance [23]. Rabbits developing a greater deformity suffered a more significant reduction in concave lung volume and function. Post-mortem studies on humans largely accord with this experimental data. Davies and Reid studied four cases with scoliosis aged between 8 and 18 years at time of death, identifying distorted lungs of reduced lung size with alveoli of reduced number, abnormal shape, and increased (emphysematous) size; these findings supported by Boffa et al. [24].

Notwithstanding this, the causal relationship of scoliosis development and resultant abnormal lung tissue is not absolutely established, with Davies and Reid questioning the histological evidence for the reduced chest volume and compression of the lung, due to scoliosis, as the cause for alveolar loss [25]. Although evidence is largely observational, it is possible that the maldevelopment of lung and spine deformity reflect aspects of the same disease, rather than the spine deformity resulting in changes in otherwise potentially normal lung tissue.

#### 3.2.2. Chest Cavity

The boundaries of the chest cavity are the ribs, sternum, vertebrae, and diaphragm. Shadowing the development of the lung tissue is the growth of this ‘rib–vertebral–sternal complex’. During growth the chest develops from the round cross section of the new-born to a more ovoid shape. Subsequent to the vast majority of alveolar development, further lung tissue expansion requires chest volume increases. Canavese et al. [26] identified a steady increase in thoracic volume; double in girls and triple in boys from age 5 until skeletal maturity. In a CT-based study, Gollogly et al. [27] demonstrated the very significant increase in thoracic volume after the majority of histological lung development, with lung volume increasing from approximately 1400 cc at age 8 to 4600 cc in males and 2700 cc in females at skeletal maturity [27]. Pulmonary function tests mirror such changes, with dramatic increases associated with puberty [28].

The increase in chest volume is due to growth of all aspects of the ‘rib–vertebral–sternal complex’, with spinal growth most studied. The thoracic spine grows most rapidly in the first 5 years (approximately 1.3 cm/year), followed by a slow phase preceding puberty (around 0.7 cm/year), and finally a further period of rapid growth until skeletal maturity (1.1 cm/year) [29]. Progressive deformity during growth reduces trunk height and alters the growth of the whole rib–vertebral–sternal complex. Mehta demonstrated in an animal study that spinal deformity induces chest deformity and vice versa [30].

Thoracic length can be affected by fusion of the thoracic spine, but the impact on lung function is unclear. Karol et al. [31] reviewed 28 cases undergoing thoracic fusion with minimal attempt at correction, mainly for congenital scoliosis with anterior–posterior surgery via thoracotomy at average age 3 years. At average age of 14 years, 12/18 had an FVC and/or FEV1 < 50% predicted. Those with a thoracic length (T1–T12) < 18 cm appeared to fare worst of all, with 10/16 having a severe restrictive lung deficit, compared to 2/8 with thoracic length 18–22 cm and 0/4 in those with >22 cm length [31]. This data have resulted in a widespread clinical aim of achieving 18–22 cm thoracic length with surgery for EOS. This simple aim, in such a heterogeneous group of patients, including non-congenital scoliosis, has reasonably been questioned including by the authors, especially given that congenital scoliosis is associated with worse PFTs than other aetiologies for similar Cobb angle [9,32]. Notably, Johnston et al. found similar percentage predicted FEV1 and FVC in those with less than or greater than 18 cm thoracic length, and highlighted that even amongst those with short thoracic length several had well-preserved PFTs [32].

Although not specifically assessing thoracic length, Goldberg et al. reported similarly chastening findings to Karol of a group of 11 children with infantile idiopathic scoliosis undergoing surgery before age 10, with mean FVC and FEV1 of 41% predicted at mean age 20 [33]. Like the series of Karol et al. [31], the majority had anterior surgery during treatment, which has an independent deleterious effect on lung function [34].

Glotzbecker et al. [35] assessed the relationship between 2D measurements of thoracic size and PFTs in 121 patients, mean age 9, who had undergone treatment for Early Onset Scoliosis of any aetiology. Absolute T1–T12 height and chest width were moderately correlated with FEV1, FVC, and TLC.

Aside from thoracic length, which dominates the scoliosis literature, little has been published regarding other aspects of the rib–vertebral–sternal complex. Thoracic lordosis results in reduced anterior–posterior diameter with associated reduction in volume [36]. Rotation of the spine is associated with rib deformities and chest asymmetry, likely resulting in both volume reductions and mechanical functional deterioration.

To conclude, simple 2D measures do not explain much of the variance in PFTs given the complexity of the respiratory system. The widespread acceptance of thoracic length as a surrogate for pulmonary function is not supported by clinical data.

#### 3.2.3. Chest Function

Campbell defined the normal thorax as having two characteristics: normal volume and the ability to change volume [18]. Chest volume change requires a mobile chest wall with functional respiratory muscles including the diaphragm and distensible lungs. Absent, fused, and/or deformed ribs will affect this chest mobility. Without congenital chest deformity, Jones et al. [37] conjectured that the alteration in relative lung volumes and rib/intercostal alignment associated with scoliosis alone led to mechanical inefficiency, noting reduced maximum expiratory pressure (MEP) in those with scoliosis compared to non-scoliotics. Reduced maximum inspiratory (MIP) and expiratory pressures in those with non-neuromuscular Early Onset Scoliosis were also observed by Redding et al. in 49 cases, with 27% and 51% having MIP and MEP > 2 S.D. less than normal [38]. Reduced pressures seemed independent of coronal deformity or surgery.

Romberg et al. [39] assessed chest wall movement on long-term follow up of treated (brace or surgery) cases of idiopathic scoliosis diagnosed < 10 years. The cohort were left with on average mild–moderate deformity (mean Cobb 35–36°, rib hump 11–16°) and reduced thoracic expansion and breathing movements. MIP and MEP were reduced in those treated with surgery but not those who were braced. However, they identified only low–moderate strength correlations between thoracic expansion and TLC/FVC/FEV1.

Most other chest movement studies relate to AIS and are therefore less relevant to this review. Jones et al. [37] noted a similar effect on PFTs with experimentally reduced chest movement in those with and without AIS, suggesting that chest wall remains mobile in AIS and chest wall changes have a limited pulmonary impact. Conversely, Leung et al. identified reduced chest and spinal movement in those with untreated moderate AIS compared to controls and strong correlations between chest movement and VC [40]. Studying functional MRI on cases with similarly moderate severity AIS identified significant reductions in chest wall motion in those with scoliosis [41].

Compliance, defined as the change in chest volume for a unit change in transthoracic pressure, is distinct from chest movement. In a study of children with TIS embarking on expansion thoracoplasty, compliance was found to be mildly reduced, with further loss of compliance following treatment [42]. Others note elevated RV:TLC ratios in those embarking on VEPTR treatment, implying reduced chest compliance [43]. Separate from chest compliance is lung compliance at lower lung volumes, in which, as seen in EOS, the lung is less distensible, requiring a greater pressure change to increase its volume.

Deformity of the diaphragm and its attachments have long been proposed as a potential influence on chest function in EOS but have not been studied in this population. Two studies using MRI were unable to identify significant differences in diaphragmatic motion in those with and without moderate AIS [41,44].

#### 3.2.4. Airways

Although EOS is associated with a restrictive lung deficit, increasingly there is recognition that airway obstruction may independently influence lung function (33–76% of EOS cases) [31,45,46]. Redding et al. [45] examined 49 children with EOS of mainly congenital and syndromic aetiology undergoing growing rod treatment, and found that three quarters had a persistent obstructive deficit, unrelated to their FVC. This worsened over a 5-year follow up in just over half, with the degree of obstruction being most related to Cobb angle at the end of follow up rather than at the start. From those identified, a sub-group of 19 were assessed with a bronchodilator of which 6 improved, inferring concomitant asthma. Surgeons should thus consider respiratory input in cases of EOS when an obstructive deficit is identified.

In the absence of asthma, the cause of obstructive deficits relates to airway compression, either directly or secondary to bronchial rotation. Midthoracic lordosis as a cause for bronchial compression was described in a range of EOS cases by Dubousset et al. [36] and has since been confirmed by other authors [46,47,48]. Mcpail et al. [46] used bronchoscopy and MRI in their series of syndromic and congenital cases, identifying compression of major airways between the lordotic rotated thoracic spine and pulmonary artery on the side of the convexity, with further impact on the left from the descending aorta. Airway compression affects predominantly the lung on the convexity, in comparison to lung volume deficits in EOS which affects mainly the concave side more greatly. Compression may result in atelectasis and recurrent pneumonia [36].

## 4. The Effect of Contemporary Surgical Management on Respiratory Function in EOS

The observed respiratory function of children treated with early fusion has motivated surgeons to pursue ‘growth friendly’ strategies. Such strategies aim to facilitate respiratory development by maximising spinal growth and controlling spinal deformity progression.

‘Growth friendly’ strategies have evolved and are currently classified based on the principle of treatment: distraction, compression or guided growth [49].

### 4.1. Distraction-Based Systems

Distraction-based systems evolved from Harrington rods to Traditional Growing Rods (TGRs) as described by Akbarnia et al. [50]. The principles of TGRs were then adopted with the use of Magnetically Controlled Growing Rods (MCGRs) which aimed to limit the number of surgeries and associated complications of TGRs, with non-invasive lengthenings in the outpatient setting. Thus, widespread international adoption of MCGRs has been seen.

#### 4.1.1. Traditional Growing Rods (TGRs)

**Figure 1:**
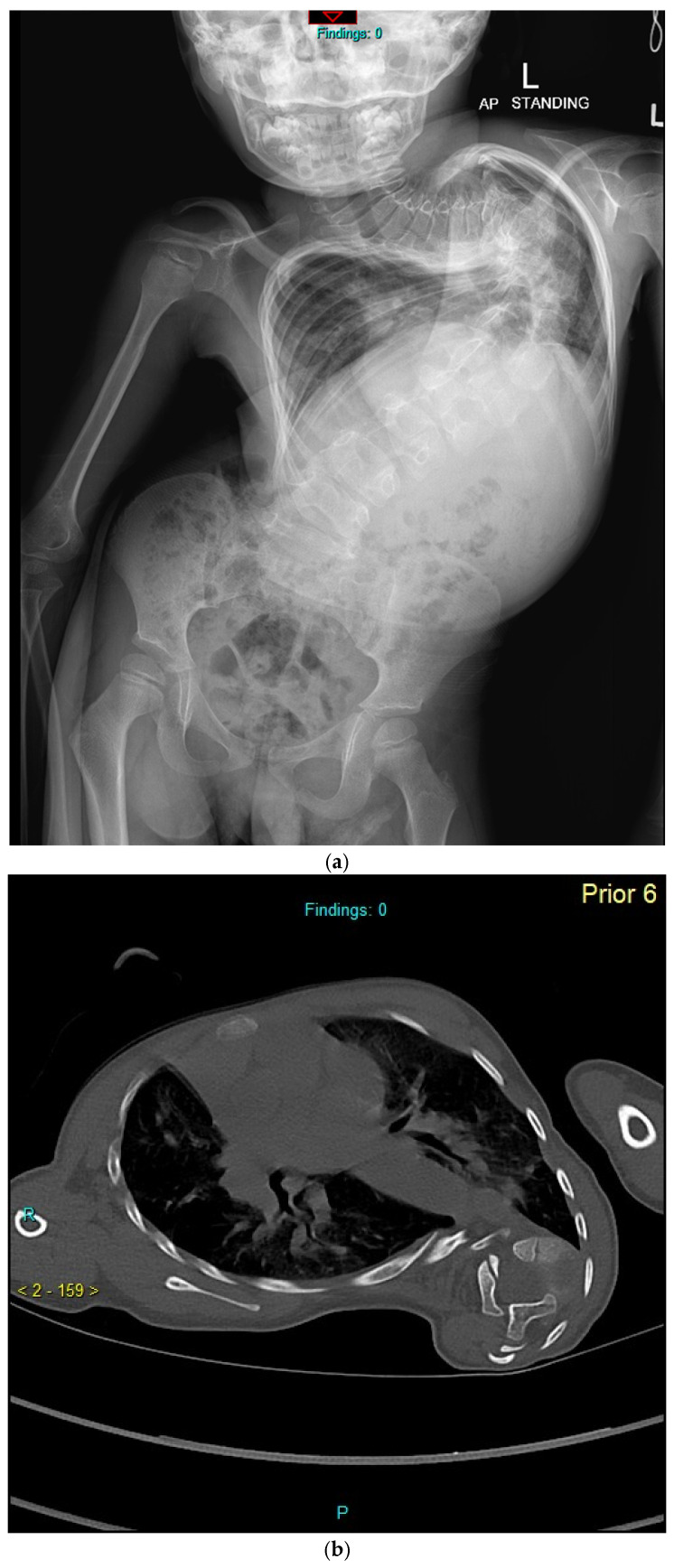

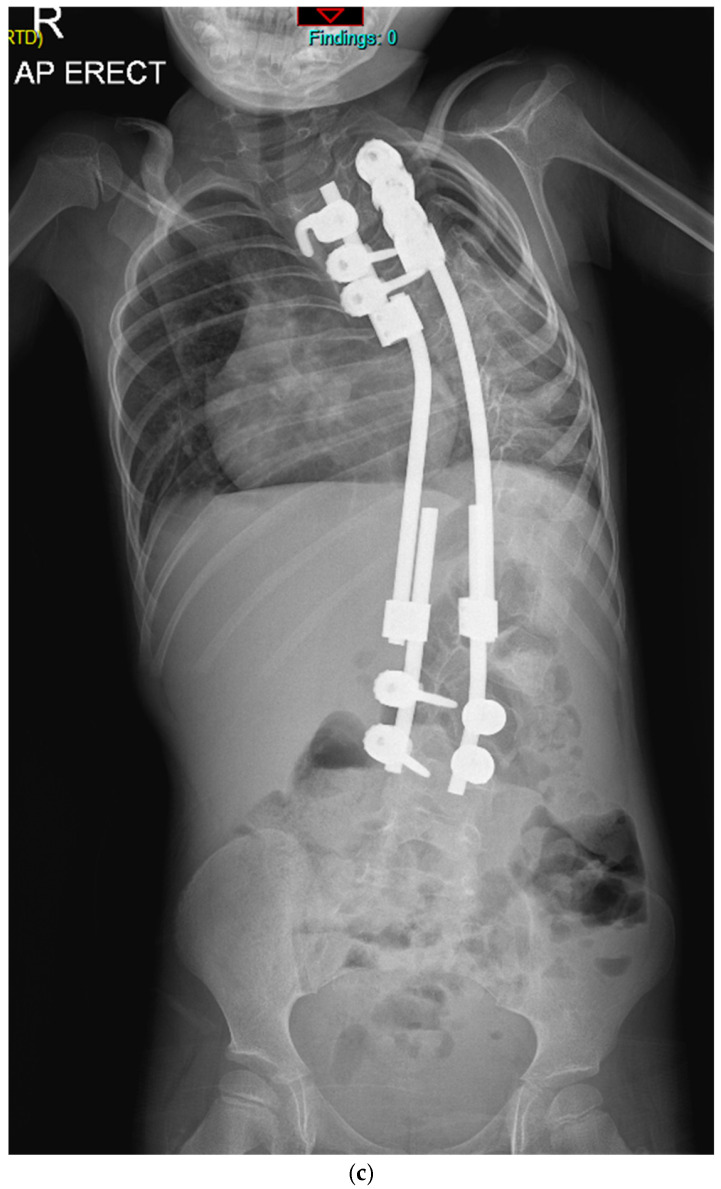

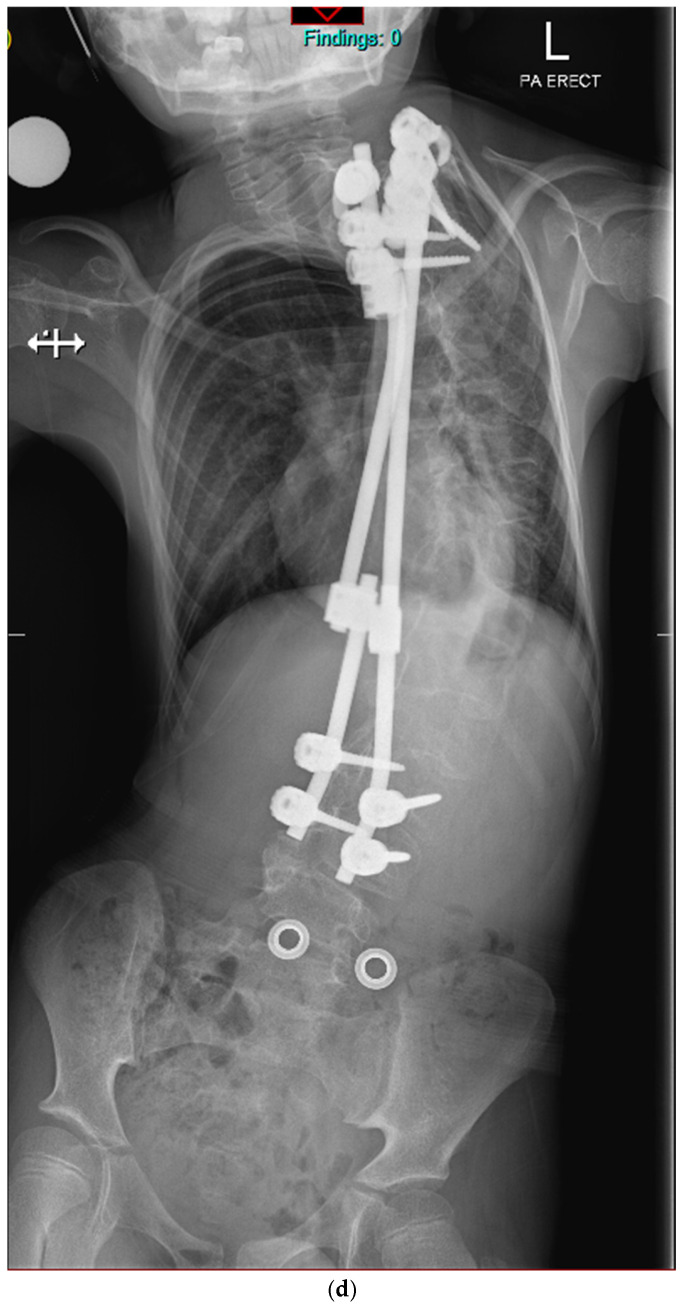

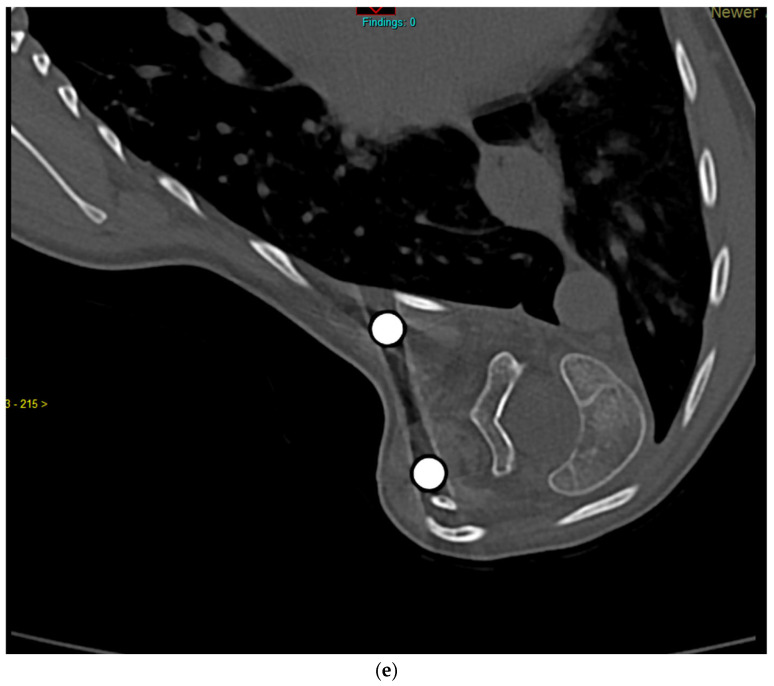
(**a**–**e**): An illustration of severe infantile onset idiopathic scoliosis presenting to our service at age 4 years 3 months with T1–T12 length 8.9 cm. (**a**): Pre-op AP X-ray. (**b**): Pre-op CT scan demonstrating severe rotation at the apex and chest deformity. At this time, sleep study identified mild sleep disordered breathing, PFTs FEV1 and FVC 25% predicted. (**c**) After halo-gravity traction, TGR inserted at age 4 years 11 months with immediate post-op improvement in T1–T12 length to 15.6 cm. (**d**) After four subsequent open TGR lengthenings, AP X-ray with T1–T12 length 19.8 cm. At age 8 sleep study again confirms mild sleep disordered breathing with FEV1 33% and FVC 31%. (**e**) Post-op CT scan demonstrates severe rotation and intrusion of the spine into the chest cavity, which has not been addressed with this technique.

TGRs are the most studied implant system in the management of EOS and are considered the gold standard (Figure 1a–e). Akbarnia and Thompson [50,51] provided the earliest evidence in favour of TGRs for controlling coronal deformity and significantly increased thoracic height at mean 4-year follow up, backed by a long-term study until final definitive fusion [52]. These studies demonstrated an effective increase in thoracic length with serial lengthening, with T1-S1 gains between 1.01 and 1.84 cm/year, depending on the initial age of instrumentation and rate of serial lengthening [50,51,52,53]. Sankar et al. noted T1-S1 height gains with TGRs were comparable to normal growth between 5 and 10 years of age, but the length gained from successive distractions declined over time as described by the ‘Law of Diminishing Returns’ [54].

Despite favourable evidence for TGRs in achieving spinal growth and deformity control, there are very few recent studies assessing pulmonary outcomes. Thoracic length is the usual surrogate marker for pulmonary function, with the limitations of this metric already discussed.

Johnston et al. [55] reported that although the absolute lung volumes increased by the time of graduation with serial lengthening, the percentage predicted tests of FEV1 and FVC remained the same or marginally decreased. Multiple other studies have noted similar findings with PFTs remaining the same or worse than pre-treatment levels by the time of graduation [56,57,58], further substantiated by the recent long-term follow up study by Redding et al. [59]. Johnston [32] noted that the children with FVC or FEV < 60% predicted prior to maturity are unlikely to avoid respiratory morbidity from TIS, regardless of T1–T12 height exceeding 18 cm and irrespective of surgical treatment. In a recent study, Johnston et al. [60] reported the PFT outcomes for patients followed up until the final graduation surgery with various growth-friendly constructs. They reported that although T1–T12 and T1-S1 height increased more significantly in TGR cohort, the PFT values remained unchanged after final fusion surgery.

#### 4.1.2. MCGRs (Magnetically Controlled Growing Rods)

Initial studies of MCGRs were encouraging, with the patients achieving T1–T12 and T1-S1 height comparable to TGRs. Akbarnia et al. [61] and Cheung et al. [62] reported mean increase in spinal height closely correlated to or sometimes exceeding the predicted spinal growth in normal subjects along with a satisfactory deformity control. With regard to the effect on respiratory function, a recent systematic review of clinical outcomes following MCGRs showed most publications had considered the thoracic spinal height as a proxy for improved PFTs with notable absence of objective measures of pulmonary function [63]. Johnston et al. reported worsening of pulmonary outcomes after the final fusion surgery when compared to pre-treatment levels in a small subset of patients with idiopathic EOS treated with MCGR [60]. Another retrospective study also noted a decrease in percentage predicted values of FEV1 and FVC at 2-year follow up in a small subset of patients [64].

Therefore, despite a of wealth of publications for MCGR outcome in EOS, the pulmonary outcomes in non-syndromic patients remain sparsely studied and largely unknown. The neuromuscular population has been better studied with several reports noting improved PFTs when managed with MCGRs [65,66,67].

#### 4.1.3. VEPTR (Vertical Expandable Prosthetic Titanium Rib)

VEPTR was developed by Campbell for the treatment of infants with congenitally fused ribs, often with scoliosis, who were in respiratory failure. The VEPTR device facilitated division of the fused rib segments from spine to sternum and vertical expansion of the chest wall, similar to putting up an umbrella. This so-called expansion thoracostomy also resulted in deformity correction and control of the spine, prompting evolution of its use to those with EOS but without true TIS [68,69].

VEPTR as a growth-friendly posterior-based distraction device for progressive idiopathic EOS without rib abnormalities was originally initiated as a prospective multicentre study in 2007 by the Paediatric Spine Study Group (PSSG). Since then, various centres have demonstrated VEPTR as an effective alternative to growing rods in the treatment of non-syndromic EOS. Although these prospective studies had a heterogeneous mix of patient data, the mid-term outcome demonstrated a significant increase in thoracic height and maintenance of deformity control [70,71,72,73,74,75].

Pulmonary outcomes with VEPTR appear similar to those with growing rods in idiopathic EOS. Though VEPTR has been demonstrated to significantly increase in the size of the concave side of the thorax, along with spinal growth, PFT data are less impressive [69]. Multiple studies have documented that the lung function tests did not change from the baseline in terms of percentage predicted FEV1 and FVC% [15,43,76,77]. Dede et al. [77] noted that despite early promise, the long-term pulmonary outcomes were adversely affected in part by implant-related complications and unplanned operations. Although Emans et al. [70] noted an increase in post-operative CT lung volumes (CTVol) following VEPTR, Mayer and Redding reported a significant decrease in FEV1%, FVC%, and static lung volume measurements of RV and TLC by plethysmography [43].

To conclude, given the multiple implant-related issues and the lack of clinically significant improvement in PFTs, VEPTR are not currently favoured in treatment of EOS.

### 4.2. Compression-Based Systems

Various compression-based systems from open hemiepiphysiodesis, open/thoracoscopic vertebral body stapling, and most recently vertebral body tethering (VBT) have been used for growth modulation in skeletally immature children. VBT has been used in older EOS children > 8 years, dubbed as ‘tweeners’, to achieve deformity control through growth modulation. Multiple studies have been published with regard to the efficacy of the use of VBT in AIS along with established good pulmonary outcomes [78,79,80,81]. However, PFT data for EOS patients treated with compression-based implants is lacking in the current literature.

### 4.3. Guided Growth Systems

Growth guidance was initially popularised by Luque et al. [82] who developed a ‘Trolley’ system, allowing sliding of multiple level sublaminar wires along guidance rods. More recent adaptations have been made. Ouellet [83] re-visited the concept with a so-called ‘Modern’ Luque trolley (MLT), with a series of 5 patients showing significant decrease in Cobb angle measurement and increase in thoracic height of 2.3 cm (0.7–4.1 cm) on average at 2.5-year follow up. A recent prospective multicentre trial [84] studied the re-operation rates for 18 patients with MLT when compared to 43 children with other growth-friendly implants (18 MCGR, 16 TGR, and 9 Rib-based implants). Despite favourable results in terms of complications and re-operations, there are no available PFT outcome data.

#### Shilla Growth Guidance System (SGGS)

McCarthy and Luhmann [85] described a novel concept of growth guidance with apical control/fusion and growth continuing via sliding screws over the proximal and distal ends of the curvature.

Short-term results showed clinically significant increase in thoracic height. A subsequent follow up study at 5 years with a cohort of 40 patients noted a significant increase in T1–T12 and T1–S1 height [86]. The PFTs of 6 patients with reproducible results showed FVC% and FEV1% > 50% with mean values > 60%, but no baseline data were available. The SAL (Space Available for Lung) determined on radiographs showed an average increase of 31% in the concavity. A follow up study showed outcomes with Shilla comparable to TGRs regarding deformity correction, thoracic, and T1–S1 height but with a significantly decreased number of surgical procedures [87]. This was contrasted by Andras et al. [88], where TGRs had a more favourable radiological outcome with the Shilla system having a higher rate of unplanned procedures and Nazareth et al. [89] where T1–S1 height increased by only 1/3rd of the predicted with Shilla with similar complication rates.

Ahmad et al. [90] described a ‘Modified Shilla Technique’ of growth guidance with active apex compression (APC) by pedicle screws on the convex side on either side of the most wedged vertebra or the apex in an extra-periosteal manner. This is a modification of the original Shilla whereby instead of apical fusion and apex correction, the apex is compressed without fusion with sliding screws proximally and distally [91]. Available data shows comparable change in spinal parameters when compared to treatment with TGR/MCGR [92,93,94]; however, presently, PFT data are lacking.

### 4.4. Hybrid Techniques

Apical control technique (ACT) has been described by Johnston et al. to achieve rotational control during repeated serial distractions with TGRs [95,96]. They postulated better rotational control with improved symmetry of the hemi-thoraces. Two papers have demonstrated favourable results (ACT with TGR/MCGR) [97,98] in terms of deformity correction, improved thoracic height, and significant improvement in apical rotation. Crucially, however, SAL, as measured in 3d CT Lung, did not improve significantly. Again, the thoracic spinal height was used as surrogate markers in these studies without any PFT data.

Skov et al. [99] described a further minimally invasive hybrid technique, which combined convex growth guided rod with apical control and concave serial distractions. The data were encouraging for deformity correction and increase in spinal parameters at 5-year follow up for 38 patients. Pulmonary function tests (FVC%, FEV1%) remained static or decreased slightly at mean 3-year follow up or at final definitive fusion.

Finally, it is notable that the literature regarding treatment of EOS is mainly categorised by different treatment modalities, rather than underlying condition and age of presentation. There are limited data, unsurprisingly, on the treatment outcomes affected by aetiology (Table 1). It may be that congenital EOS fares worse off from a respiratory standpoint than other aetiologies [59]. Reports suggest neuromuscular cases have been shown to improve their respiratory function with surgical treatment [65,66,67]. Future studies should further develop this, reporting outcomes focused on EOS aetiology and age of presentation rather than treatment type.

## 5. Conclusions

EOS clearly has an adverse effect on pulmonary function but the complex interrelationship between the spine, chest, pulmonary structures, and their development makes understanding and quantification challenging. It is, however, well established that if left untreated EOS results in premature death with poor pulmonary outcomes, with early fusion surgery failing to address this. This has led to the adoption and evolution of so-called growth-friendly surgical techniques to manage EOS each with pros and cons. Outcomes from such surgical interventions have been dominated by simple radiological measurements of deformity and thoracic height often being used as surrogate measurement of pulmonary function. Assessing pulmonary function in this group of young children is challenging and presently there is a paucity of studies assessing the specific effect of growth-friendly surgeries on this. Available evidence suggest that the current surgical strategies at best maintain pulmonary function at pre-treatment levels. Ultimately, the most realistic and long-term goal in management strategy of EOS appears to be to prevent worsening and maintain pulmonary function at an acceptable level until final graduation surgery.

## Figures and Tables

**Table 1:** Summary of existing studies of EOS treated with growth-friendly implants reporting pulmonary functions post-operatively.

Author, Year	Study Design, N	EOS Patient Sub-Group	Growth-Friendly Implant Used	Cobbs Angle (°) Pre and Post Treatment at Final Follow Up	T1–T12 Height–Pre and Post Treatment (cms) at Final Follow Up	Mean Follow Up	PFT Measurements Pre-op (Mean)	PFT Measurement–Post-op/Final Fusion (Mean)	PFT Outcomes
Johnston et al., 2017 (JBJS) [55].	Prospective, N = 12 Until Final Graduation	Idiopathic—3 Congenital—3 Syndromic—2 Neuromuscular—4	TGR—10 VEPTR—2	88 pre-op to 47 post-op (Mean)	13.3 pre-op to 22.3 post-op (mean)	33 months	FEV1%—53.8 FVC%—53.5	FEV1%—52.1 FVC%—55.3 (Post Graduation)	No statistically significant improvement at graduation
Chang et al., 2021 (J. Child Orthop) [56].	Retrospective, N = 17	Congenital—8 Idiopathic—4 Neuromuscular—2 Syndromic—1 Combined—2	TGR	65 pre-op to 43 Post-op (Mean)	14.2 pre-op to 18.6 post-op (mean)	6.2 years (Minimum 2-year fup post-op)	FEV1%—50 FVC%—51	FEV1%—53 FVC%—55	No statistically significant improvement
Celebioglu et al., 2020. (J. Ped. Orthop) [57].	Retrospective, N = 8 Age-matched comparative study	Idiopathic—8	TGR	66 pre-op to 40.5 (mean) post-op in TGR group	23.8—TGR vs. 25.2 AIS after treatment	Followed up until graduation to final fusion	No pre-op data provided, compared with control and AIS groups post-op	FEV1%—72.5 FVC%—72 (Post Graduation)	Statistically reduced scores when compared with control and AIS group
Yang et al., 2025 (JBJS) [59].	Retrospective, N = 51	Syndromic—23 Congenital—1 Idiopathic—6 Neuromuscular—6 Thoracogenic—5	VEPTR—31 TGR—9 MCGR—11	77.1 pre-op to 60.6 post-op (Mean)	16.0 pre-op to 18.6 post-op	Graduation—N = 23 PFT Median duration-44 months	FEV1%—53.47 FVC%—54.20	FEV1%—50.74 FVC%—52.80	No statistical significance except in congenital scoliosis where there is further pulmonary decline at graduation
Johnston et al., 2025 (JBJS) [60].	Retrospective, N = 51	Idiopathic-15 Syndromic—11 Congenital—7 Neuromuscular—18	TGR—24 Vs MCGR—27	81 vs. 89 pre-op (MCGR vs. TGR) to 32 vs. 50 post-op (MCGR vs. TGR)	16.3 pre-op to 21 post-op MCGR; 14.8 pre-op to 21.6 post-op TGR	Follow up until after graduation	MCGR: FEV1%—64.63 FVC%—66.31 TGR: FEV1%—47.1 FVC%—44.5	MCGR: FEV1%—49.29 FVC%—55.29 TGR: FEV1%—56.83 FVC%—60.1 (Post Graduation)	Significant decrease in MCGR group; no statistically significant improvement in TGR
Munigangaiah et al., 2024 (Med Res. Archives) [64].	Retrospective, N = 8	Idiopathic—8	MCGR	71 pre-op to 35 post-op (Mean)	17.5 pre-op to 20 post-op (Mean)	Minimum 2-year post-op follow up	FEV1%—82.26 FVC%—79.5 FEV1 z score—1.5 FVC z score—1.7	FEV1%—72.4 FVC%—71.07 FEV1 z score—2.4 FVC z score—2.4	Statistically significant decrease in predicted volumes and z-scores
Yoon et al., 2014 (Spine) [66].	Retrospective, N = 6	Neuromuscular—6	MCGR	87 pre-op to 34 post-op (Mean)	Mean lengthening of 2.49 at 2-year follow up	2.5 years	FEV1%—27 FVC%—27	FEV1%—45 FVC%—41	Statistically significant increase in lung functions
Motoyama et al., 2005 (Spine) [15]	Prospective, N-10	Congenital—5 Syndromic—2 Combined—3	VEPTR	49.7 Pre-op to 35.3 Post-op (Mean)	N/A	FVC% measured under GA, during each subsequent lengthenings; mean fup—22 months	FVC%—69.2 MEF10—0.21	FVC%—70.2 MEF10—0.23	No statistical difference before and after lengthenings
Gadepalli et al., 2010, (J. Ped surg) [76].	Prospective, N-26	Congenital—12 Neuromuscular—5 Syndromic—2 Thoracogenic—7	VEPTR	64.7 pre-op to 46 post-op (mean)	N/A	PFT at 6-months post-op 3DCTR at 1 yr post-op	FEV1%—54.6 FVC%—58.1 3DCTR Lung Vol—944.2	FEV1%—51.8 FVC%—55.9 3DCTR Lung Vol—1042.1	No statistical difference before and after lengthenings
Dede et al., 2014 (JBJS) [77].	Retrospective, N-21	Congenital—11 Syndromic—6 Neuromuscular—3 Idiopathic—1	VEPTR	80 pre-op to 67 post-op (Mean)	12.3 Pre-op to 14.9 post-op	6-years mean follow up, FVC% measured under GA	FVC%—77 SAL—0.77	FVC%—58 SAL—0.87	Statistically significant decline in pulmonary functions at final follow up
Mayer et al., 2009 (J. Pediatr. Orthop) [43].	Retrospective, N = 53, multicentre analyses	Undefined EOS	VEPTR	57.9 pre-op to 46.5 post-op	N/A	7.7 +/− 4.8 months	FEV1%—58.9 FVC%—61.5	FEV1%—52.1 FVC%—54.3	Statistically significant decrease in lung functions
Emans et al., 2005 (Spine) [70].	Prospective, N = 31	Congenital/Syndromic Scoliosis with fused ribs and TIS-31	VEPTR	55 pre-op to 39 post-op (Mean)	Mean of 2.3 cm increase immediately vs. 1.2 cm at 1-year follow up	Minimum 2-year post-op follow up	FEV1%—72.9 FVC%—71.6	FEV1%—78.6 FVC%—71	No statistically significant difference in PFTs
Skov et al., 2020 (International Orthop) [99].	Prospective, N = 38, N = 14 for PFT	Idiopathic—6 Neuromuscular—5 Syndromic—2 Thoracogenic—1	CB + CB/MCGR at concave)	76 pre-op to 42 post-op (mean) at final follow up	19 pre-op to 23.8 post-op (mean)	Minimum 1-year follow up post-op	FEV1%—66 FVC%—62	FEV1%—62 FVC%—58	No statistically significant difference in PFTs

Legends: EOS—Early Onset Scoliosis; TGR—Traditional Growing Rods; MCGR—Magnetically Controlled Growing Rods; VEPTR—Vertical Expandable Prosthetic Titanium Rib; FVC%—Percentage predicted Forced Vital Capacity; FEV1%—Percentage Predicted Forced Expiratory Volume in 1s; 3DCTR—3-dimensional reconstructions of thoracic CT scans; MEF10—Maximum Expiratory Flow at 10% of FVC; SAL—Space Available for Lung; CB—Cody Bunger Concept; N/A—Not Available.

## Data Availability

Data can be made available on request.

## Abbreviations

The following abbreviations are used in this manuscript:

EOS	Early Onset Scoliosis
FVC	Forced Vital Capacity
VC	Vital Capacity
TLC	Total Lung Capacity
FEV1	Forced Expiratory Volume in 1s
TIS	Thoracic Insufficiency Syndrome
MRI	Magnetic Resonance Imaging
PFT	Pulmonary Function Tests
MEP	Maximum Expiratory Pressure
MIP	Maximum Inspiratory Pressure
AIS	Adolescent Idiopathic Scoliosis
RV	Residual Volume
VEPTR	Vertical Expandable Prosthetic Titanium Rib
TGR	Traditional Growing Rods
MCGR	Magnetically Controlled Growing Rods
Cm	Centimetres
CTVol	Computed Tomography Lung Volumes
VBT	Vertebral Body Tethering
MLT	Modern Luque Trolley
PSSG	Paediatric Spine Study Group
SAL	Space Available for Lung
FVC %	Percentage Predicted Forced Vital Capacity
FEV1%	Percentage Predicted Forced Expiratory Volume in 1s
APC	Active Apex Compression
ACT	Apical Control Technique
